# Meta-Analysis and Systematic Review of Neural Stem Cells therapy for experimental ischemia stroke in preclinical studies

**DOI:** 10.1038/srep32291

**Published:** 2016-08-24

**Authors:** Lukui Chen, Guilong Zhang, Yuchun Gu, Xiaoyuan Guo

**Affiliations:** 1 Department of Neurosurgery, Zhongda Hospital, School of Medicine, Southeast University, Nanjing, China; 2 Molecular Pharmacology Laboratory, Institute of Molecular Medicine, Peking University, Beijing, 100871, China

## Abstract

To evaluate the preclinical studies using NSCs transplantation therapy for experimental ischemic stroke, and determine the effect size of NSCs therapy and the correlations between different clinical measures. We firstly searched literatures to identify studies of NSCs therapy in animal cerebral ischemia models, and then calculated the quality score of studies, assessed the effect size of NSCs therapy relative to behavioral and histologic endpoints by meta-analysis. A total of 37 studies and 54 independent treated interventions were used for systematic review and meta-analysis. The median quality score was 5 of 10. 36 studies (53 intervention arms) reported functional outcome, 22 studies (34 intervention arms) reported structural outcome. After adjusted by subgroup and sensitivity analysis, the mean effect sizes were improved by 1.35 for mNSS, 1.84 for rotarod test, 0.61 for cylinder test, and 0.84 for infarct volume. Furthermore, effect size had a certain interaction with clinical variables, for example early NSCs therapy *etc.* In this preclinical studies, we demonstrated that transplanted NSCs significantly improved outcomes (both functional and structural outcome) in ischemic stroke. It is suggested that future preclinical animal model studies of stroke should improve study quality validity and reduce potentially confounded publication bias.

Ischemic stroke, also known as cerebrovascular accident, is caused by the decreased or interrupted blood supply in part of the brain. It leads to the dysfunction in part of brain tissue causing long-term disability and high rate mortality^1^,^2^. Currently there is no efficient therapy to sufficiently improve clinical recovery after stroke. tPA (tissue plasminogen activator) is the most popular treatment drug in clinical. As a thrombolytic drug, it plays a major role in the early stage of ischemia. However, it has a very short time window (less than 6 hours) and can increase the risk of cerebral hemorrhage^3^. In addition, only approximately 5% of stroke patients receive this treatment across the United States^4^. Abundant evidences suggest that restorative therapies, including stem cell therapy, have the best potential to reduce neurological disability^5^,^6^,^7^.

Recent advances in stem cell research showed that both endogenous and exogenous NSCs could raise promising therapies. However endogenous neuro-regeneration has been depicted that it is insufficient to repair neural tissue of injured brain^8^,^9^. A number of studies confirmed that NSCs-based transplantation therapy for ischemia stroke has been successfully carried out^7^,^10^,^11^. The results confirmed that transplanted NSCs could significantly facilitate brain tissue repair and neurological functional recovery. The main strategies of NSCs treatment include reducing neuronal apoptosis, improving the microenvironment of ischemic areas and promoting functional recovery of damaged neurons. Furthermore, evidences further suggest that the transplanted NSCs are not only associated with new neurons or glial cells renewal (via cell replacement), but also expected to change the diseased tissue milieu. The NSCs are expected to mediate homeostasis and tissue repair through regulating brain trophic factors secretion or interacting with CNS-resident and CNS-infiltrating immune cells^8^,^12^. Thus NSCs exert direct neuroprotection function in surrounded ischemic sites and the further modification of NSCs fate can repair neuronal network^13^. In addition, NSCs have been regarded as an excellent safety source in clinical application of human ischemia stroke.

Meta-analysis is a statistical overview of the results^14^,^15^. By using a statistical approach to combine the results from multiple studies over individual studies, it improves the estimate of the size of effect and resolves uncertainty^16^. There are many studies and interventional methods for the use of NSCs in experimental stroke, while a global estimate of efficacy for NSCs treatment in stroke models is scarce. Therefore, the purpose of the current study is to evaluate the potential of NSCs transplantation therapy for ischemic stroke in preclinical studies.

The current meta-analysis and systematic review focus on one specific cell type (NSCs) therapy for one specific clinical disease (ischemia stroke). Firstly, based on recent Stroke Therapy Academic Industry Roundtable (STAIR) recommendations and NIH workshop^17^,^18^, the quality score of the studies was calculated. Secondly, the effect size of NSCs treatment was examined in both behavioral and histologic outcomes. Several lower quality or inadequate preclinical studies were removed to avoid overestimate efficacy and heterogeneities. Thirdly, the relationship between study quality and each effect size was checked. The robustness of NSC efficacy was examined across clinical measures of interest such as type of NSCs source and time of administration relative to stroke onset, *etc*. In conclusion, this study used systematic review and meta-analysis approaches to identify the possible publication bias and to investigate the impact of various aspects of reporting outcomes in preclinical ischemia stroke studies^19^.

## Methods

### Search Strategy

We searched the literatures in PubMed, Ovid and ISI Web of Science for animal models of cerebral ischemia until August 2015. The search strategy was (neural stem cell or NSC* or neural precursor cell or NPC*) AND (brain ischemia or stroke or cerebrovascular or middle cerebral artery or MCA* or anterior cerebral artery or ACA*), and the details are described in the online Supplementary Table 1. We excluded studies that the stroke model was hemorrhagic rather than ischemic, transgenic studies or neonatal hypoxia/ischemia models. Finally, studies that the intervention was given with tracked imaging rather than improving outcome or the NSC therapy interacted with additional biological effects such as gene modification were excluded.

### Data Extraction

We extracted these data including authors, year of publication, type of NSCs intervention, species of animal, type of cerebral ischemia, number of animal, intervention dose, time of administration, route of delivery, anesthetic used, immunosuppression and outcome assessment (functional and structural), outcome measure used, mean outcome, SD (standard deviation) or SE (standard error), number of animals per group, and study quality assessment (see online Supplementary Tables 2 and 3). We defined the treatment comparison as the outcome in control (vehicle) group compared to treated group. If there is >1 interventions were given in one study, we regarded these to be another intervention. Furthermore, if the treatment was administered in multiple doses, we considered the sum of all doses administered in the first 24 hours. Moreover, if the data from multiple brain slices were reported in structural outcomes, we only considered the infarct volume from the largest infarct in control slices. Finally, if the functional outcomes were reported for >1 time points, we only included the last time of assessment. Quantitative data were extracted from all available sources in each paper, including text and graphs. When data were only presented graphically, we measured values for mean and SD from graphs using quantitative methods on highly magnified images (GetData Graph Digitizer, version 2.26).

### Quality Assessment

Quality Score estimating method defined 10 criterias based on STAIR guidelines^17^,^20^ and was used for each preclinical study to meta-analysis and review the animal data from experimental studies checklist^21^,^22^. The criterias include: (1) publication in a peer-reviewed journal, (2) statements describing control of temperature, (3) random assignment of animals to treatment group, (4) allocation concealment, (5) blinded outcome assessment, (6) avoidance of anesthetics with known marked intrinsic neuroprotective properties, (7) use of animals with relevant comorbidities, (8) inclusion of a sample-size calculation, (9) statement of compliance with animal welfare regulations, and (10) inclusion of a statement declaring presence or absence of any conflicts of interest. One point was given for each criterion reported. Potential score ranges from 0 to 10, with higher scores indicating greater methodological rigor.

### Data Analysis

The endpoint effect size of NSCs therapy was determined as the improvement in outcome in treated (intervention) animals relative to untreated ischemic (control) groups. We used DerSimmonian and Laird random-effects meta-analysis to analyze substantial heterogeneity of studies. For infarct volume and neurological outcomes, we used standardized mean difference (SMD) approach in units of SD to report the improvement in treated groups. The effect size of last time point was used for meta-analysis. If >1 functional outcome was reported for the same cohort of animals at the same time point, we combined these in a fixed-effects meta-analysis to give a summary estimate of efficacy.

### Statistical Analysis

We examined mean effect size, 95% confidence intervals, forest plots, and significance using inverse-variance method and standard mean differences in Review Manager 5.3. Because substantial heterogeneity was present across endpoints, the random effects models were used.

We used stratified analysis with partitioning of heterogeneity to test the extent to which study characteristics explained differences in reported efficacy. Subsequently, sensitivity analysis and subgroup analysis were employed to detect the inappropriate treatment arms and further determine the heterogeneities of the rest studies. This approach can test whether studies are drawn from the homogeneous population and identify why other estimates in strata are significantly different.

For bivariate analysis, we chose 5 clinical variables of interest to examine its relation to quality score and each effect size using nonparametric method. The 5 clinical variables of interest were: (1) type of NSCs source, (2) intervention NSCs dose, (3) route of delivery, (4) time of administration relative to stroke onset, and (5) use of immunosuppression. For 2 dominant effect sizes (mNSS and infarct volume) with the largest number of published articles, data was also analyzed by multiple regression (forward stepwise; *p* = 0.1 to enter the model, *p* = 0.15 to leave) in SPSS 21.0. The type of NSCs intervention and route of delivery were converted to categorical variables for multiple regression modeling. The potential publication bias was assessed by using Funnel plots and the global efficacy was calculated. Because the process of pooling data from different behavioral outcomes measured at the same time point would confound the analysis of publication bias, we used all data rather than pooled data and resulted in a different global estimate of efficacy in the publication bias analysis.

## Results

### Study Characteristics

We identified a total of 37 studies and 54 independent treated interventions out from 2524 publications for systematic review and meta-analysis (see online Supplementary Fig. 2 and Supplementary Table 2). According to selection ctriterias, 2487 studies were excluded. We found that outcomes (functional or structural) were reported in 53 treated interventions of 36 studies and 34 treated interventions of 22 articles, respectively.

The type of cerebral ischemia was transient in majority studies and induced by using the intraluminal occlusion of middle cerebral artery (MCA). Three studies were permanent occlusion and one study was induced via thrombosis. Under different treated interventions, 39 of 54 treatments were injected directly with NSCs (including ESC or iPSC–derived NSC/NPC) and the other 15 treatments were transplanted with other cytokines/molecules (*e.g.* NGF, VEGF, hNT3, GDNF *etc.*). Furthermore, 37 of 54 interventions used rat/mouse NSCs and 17 of 54 used human NSCs. Regarding the route of delivery, 43 of 54 treatment arms were performed via intracerebral injection (stereotactic), 10 of 54 were delivered by intravenous administration (6 via tail vein and 4 via femoral vein) and 1 of 54 was injected via lumbar puncture. Anesthetic was used in 26 studies and 38 interventions during stroke induction. The use of immunosuppression had been reported in 18 of 37 articles, of which there were no reports about the use of immunosuppression in 7 interventions.

### Quality Score

The median Quality Score checklist items scored was 5 out of 10 (interquartile range, 4–6; see online Supplementary Table 4). All studies have been published in peer-reviewed journals. 22 of 37 studies reported describing control of temperature, 17 of 37 studies reported randomized allocation to treatment group, 11 studies reported allocation concealment during the experiment, 20 studies reported blinded assessment of outcome, 32 studies used avoidance of anesthetics with known marked intrinsic neuroprotective properties, none of them used animals with relevant comorbidities (*e.g*. hypertension) or reported a sample size calculation, 35 studies stated compliance with animal welfare regulations, and 12 studies stated possible conflicts of interest.

### Meta-analysis and effect size

The mean neurological score was improved by 1.91 SMD in 36 of 37 experiments (95% CI, 1.55–2.26, see online Supplementary Fig. 1A; 36 studies and 53 comparisons; 472 animals in control groups versus 537 animals in intervention groups). However, the heterogeneity between studies was significant (*χ*^2^ = 498.02; *I*^2^ = 90%; *df* = 52; *P* < 0.00001). After stratification by intervention, it showed significant improvement in outcomes. 8 out of 53 interventions were greater than 3 SMD, with one SMD of 11.29. We further evaluated the appeared most frequently neurobehavioral across the animals’ outcomes and the effect size of NSC therapy was assessed under 3 points (Table 1): (1) modified Neurological Severity Score (mNSS), (2) rotarod test, and (3) cylinder test.

**Figure 1:**
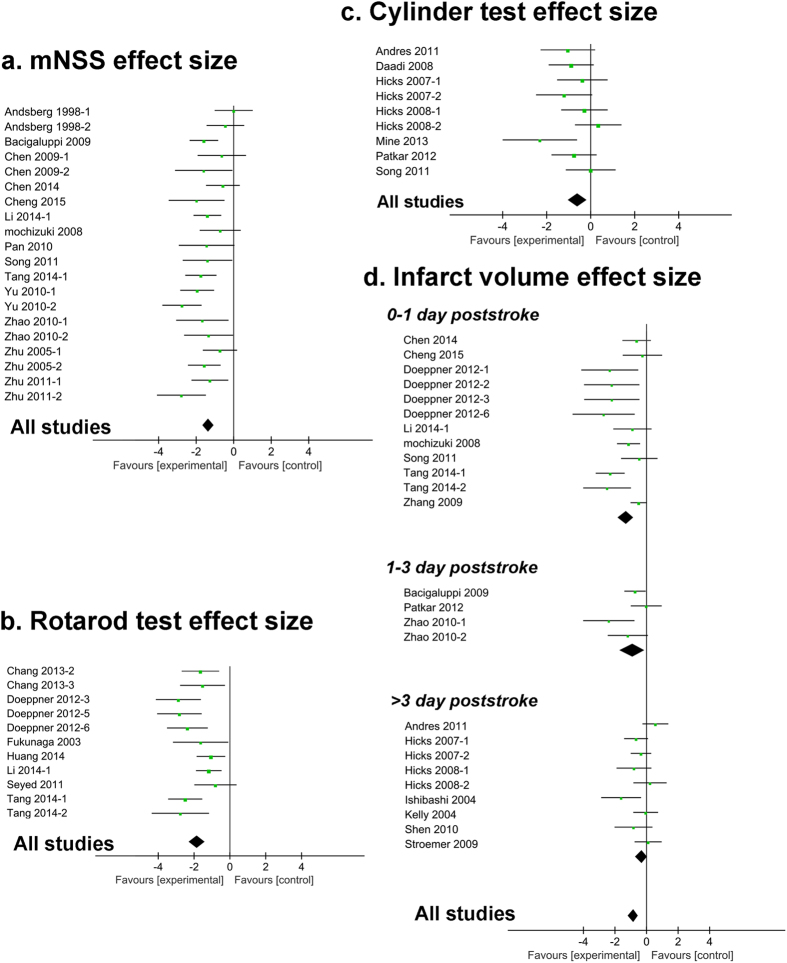
Each effect size of NSCs across studies. Forest plot showed median effect size and 95% CI for (**a**) mNSS, (**b**) Rotarod test, (**c**) Cylinder test and (**d**) infarct volume.

The effect size of mNSS was 2.0 (95% CI, 1.55–2.46; 19 studies and 28 comparisons, Table 1) and the heterogeneity was moderate (*χ*^2^ = 111.49; *I*^2^ = 76%; *df* = 27; *P* < 0.00001, see online Supplementary Fig. 1B). Although there were 8 treatment arms which effect size greater than 3.0, the heterogeneity of studies significantly went down (*χ*^2^ = 32.9; *I*^2^ = 42%; *df* = 19; *P* < 0.00001, Fig. 1a) when sensitivity analysis was performed and 8 inadaptable treatments were removed. The effect size of rotarod test was 2.48 (95% CI, 1.65–3.32; 11 studies and 19 comparisons, Table 1) with high heterogeneity (*χ*^2^ = 171.29; *I*^2^ = 89%; *df* = 18; *P* < 0.00001, see online Supplementary Fig. 1C). However, there are 2 outcomes were dominated by control groups and 6 of 19 comparisons were greater than 3.0 with the biggest effect size of 9.64. After removing those 8 inadaptable comparisons under sensitivity analysis, the heterogeneity was low to *I*^2^ = 47% (Fig. 1b). The effect size of cylinder test was 0.61 (95% CI, 0.17–1.04; 7 studies and 9 comparisons, Table 1). There was no intervention greater than 3.0 with low heterogeneity (*χ*^2^ = 10.28; *I*^2^ = 22%; *df* = 8; *P* = 0.006, Fig. 1c).

Infarct size was improved by 1.45 SMD (95% CI, 1.01–1.88; 22 studies and 34 comparisons, Table 1; 331 animals versus 308 animals in control and intervention groups) in 22 of 37 experiments, and again the substantial heterogeneity was significant (*χ*^2^ = 152.71; *I*^2^ = 78%; *df* = 33; *P* < 0.00001, see online Supplementary Fig. 1D). After stratified analysis by interventions outcomes, 9 of 34 interventions were greater than 3 SMD with the biggest SMD of 10.48 and 16.49 in one study. We further divided them into 3 subgroups according to the time of NSCs administration: 0–1 day poststroke, 1–3 day poststroke, and >3 day poststroke. After removing those 9 improper interventions with effect size over 3 SMD, the heterogeneity of each subgroup was *I*^2^ = 59%, *I*^2^ = 54%, *I*^2^ = 38%, respectively (Fig. 1d).

### Quality Score and effect size

**Figure 2:**
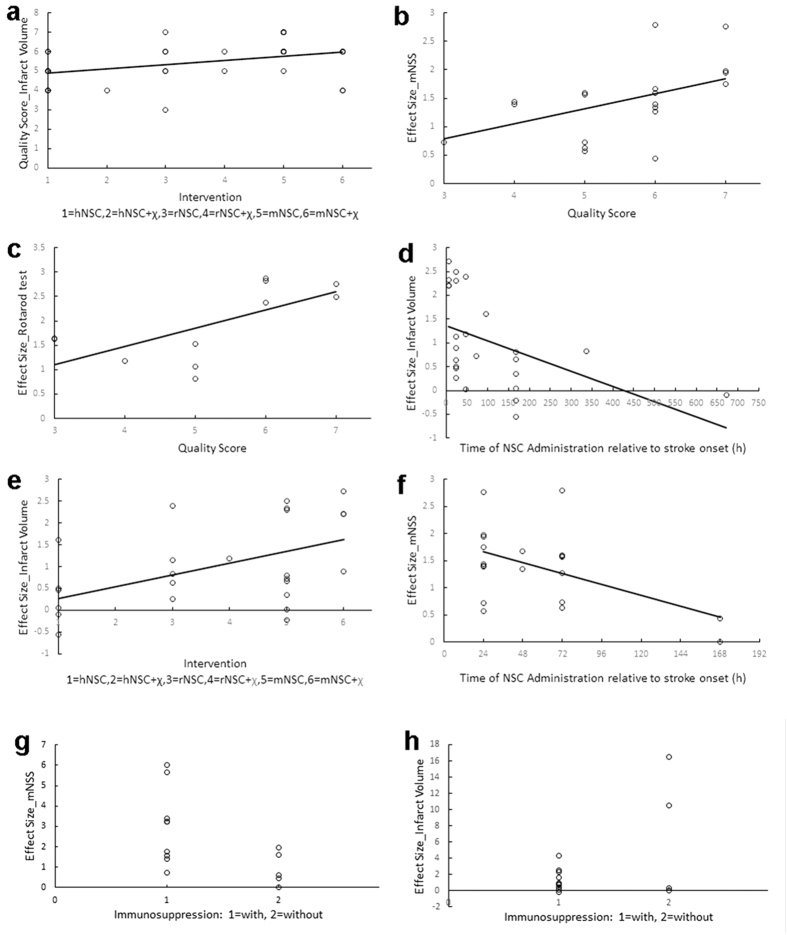
The correlations of effect size and study quality or clinical variables across studies. Bivariate analyses confirmed that study quality was little related to NSCs interventions in infarct volume (**a**), and the effect size of mNSS and Rotarod test were significant positive correlation with study quality (**b,c**). Furthermore, the effect size of infarct volume or mNSS across studies has a certain correlation to clinical variables type of NSCs source (**e**) and time of NSCs administration relates to stroke onset (**d,f**). (**g,h**) The correlations of the effect size (mNSS and infarct volume) and the use of immunosuppression in 9 reported studies (14 interventions), respectively (mNSS: p = 0.038, Infarct Volume: p = 0.048). hNSC (rNSC or mNSC) = human NSC (rat or mouse NSC); hNSC + *χ* = human NSC + cytokines (*e.g.* VEGF).

The Quality Score of all studies was not correlated (p > 0.1) to any of the 5 clinical variables of interest (NSCs source, route, time, dose and immunosuppression). Furthermore, the 5 clinical variables of interest were not related to the quality scores of dominant mNSS or infarct volume groups. However, the quality score of infarct volume was correlated with interventions (type of NSCs source, p = 0.027, r = 0.38, Fig. 2a).

We subsequently evaluated the relations between quality score and each effect size of functions (mNSS, Rotarod and Cylinder test) or structure (infarct volume). We found that there was no direct interaction among infarct volume or cylinder test with quality score. However, the quality score was significantly correlated with the effect size for mNSS (r = 0.535, p = 0.018, Fig. 2b) and the effect size for Rotarod test (r = 0.654, p = 0.029, Fig. 2c), indicating that higher study quality leads to the greater improvement in behavioral recovery associated with NSCs treatment.

### Clinical variables and effect size

Next we used bivariate analysis to reveal the relation of some interesting clinical variables and the effect sizes. Five clinical variables of interest (different NSCs intervention, intervention dose, route of delivery and time of administration relative to stroke onset, the use of immunosuppression) and two mainly effect size (mNSS and infarct volume) were analyzed, respectively. Several clinical variables were correlated with mNSS and infarct volume (Table 2). Similarly, multiple regressions (under stepwise forward modeling) reached the concordant results with bivariate analyses.

For infarct volume effect size, the time of NSCs administration (r = −0.472, p = 0.017, Fig. 2d), type of NSC source (r = 0.465, p = 0.019, Fig. 2e) and use of immunosuppression (p = 0.048, Fig. 2h) were significant related and served as significant predictors. This indicates that greater outcomes come from early NSCs intervention, *i.e.* first 24 hours poststroke and larger effects come from transplantation of homologous NSCs, *i.e.*, mNSCs for mouse or rNSCs for rats.

For mNSS effect size, the time of NSCs administration (r = −0.508, p = 0.022, Fig. 2f) and use of immunosuppression (p = 0.038, Fig. 2g) were served as the significant predictors and correlated to effect size for mNSS. It also showed that bigger effects and greater behavioral were gained from NSCs early intervention, *i.e.*, first 24 hours poststroke and transplantation of homologous NSCs.

### Funnel plots and Publication Bias

**Figure 3:**
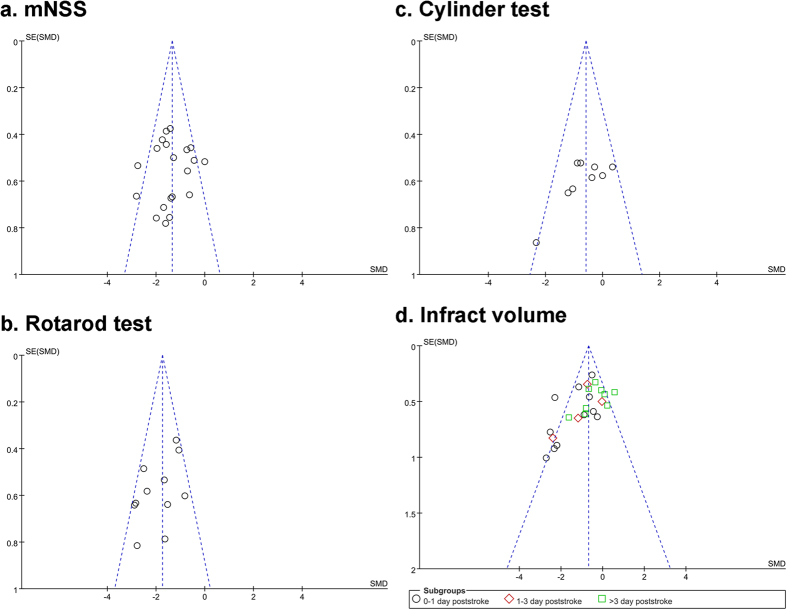
Funnel plots for neurobehavioral (**a–c**) and infarct volume (**d**) showed the distribution of researched study outcomes (circles and squares) to estimate potential publication bias.

Funnel plots were used to examine the effect size (3 behavioral measures and infarct volume, Fig. 3a–d) in relation to studies and to check the interaction of each study with publication bias to estimate study qualities and heterogeneities. Because our study has very larger effect sizes than other studies^21^,^22^ both mean values in functional and structural outcomes and the heterogeneities of studies suggested significant publication bias. After adjusting these effect sizes by subgroup and sensitivity analysis, the mean effect sizes still remained slightly high (1.35 for mNSS, 1.84 for rotarod test, 0.61 for cylinder test, and 0.84 for infarct volume, Table 1).

## Discussion

In the recent two decades, different types of stem cell have been widely investigated and applied for ischemia stroke therapy, especially with a sharp increase in NSCs and MSCs (marrow mesenchymal stem cells) transplantation^21^,^22^,^23^,^24^,^25^. Here we have searched NSCs therapy for experimental ischemia stroke and extracted data from 37 publications and 54 treatment arms for meta-analysis and systematic review. Our purpose is to find less publication bias and more robust evidence for narrative reviewing whether NSCs are likely to be useful tool for ischemia stroke therapy. This meta-analysis dominantly examined preclinical studies of NSCs in the treatment of animal ischemic stroke and found that transplanted NSCs significantly improved outcomes (both functional and structural outcome) with significant effect sizes. We further determined that effect sizes were strongly related to the time of NSCs administration relative to stroke onset and type of NSCs source (immunogenicity). However, no relevancy was discovered for the dose of NSCs and route of delivery, maybe due to most studies delivered NSCs to the lesion via stereotactic injection. Nevertheless, our data suggests further translational studies of NSCs for treating ischemic stroke in humans.

Using same Quality Score checklist items, the median quality score of current study was 5 (interquartile range, 4–6), which is higher than the study of Lees *et al*.^21^ on preclinical stem cell therapy. However, this is slightly lower than the study of Vu *et al*.^22^ on preclinical mesenchymal stromal cells for ischemic stroke. Generally, higher study quality yields more efficient evaluation on preclinical study effects^26^ and lower quality study normally overestimates intervention or experimental effects^27^. The quality score of this study was moderate and positive correlated with larger neurobehavioral outcomes (*e.g.* mNSS and Rotarod test) after NSCs administration.

However, in the current meta-analysis, each improved effect size was very high. The neurological score was increased by 1.91 SMD for studies (36 studies and 53 treatment arms), 1.45 SMD for infarct volume (22 studies and 34 treatment arms), 2.00 for mNSS (19 studies and 28 treatment arms), and 2.48 for rotarod test (11 studies and 19 treatment arms). To our surprise, there were many effect sizes of interventions were greater than 3.0, with several even more than 10.0. The big effect sizes for both structural (infarct volume) or behavioral (mNSS and rotarod test) were similar to the findings by Janowski *et al*.^28^ and Vu *et al*.^22^. Because substantial heterogeneities were existed in behavioral and anatomical gains, we adjusted those heterogeneities and potential publication bias by subgroup and sensitivity analysis. After removing those inadaptable treatments with effect sizes >3, the rest effect sizes still remained slightly high (0.84 for infarct volume, 1.35 for mNSS and 1.84 for rotarod test).

Generally, an effect size >0.8 equates is considered as a large effect^27^. We checked those treatments which effect sizes >3, and found that there were 9 intervention arms for infarct volume, 8 arms for mNSS, and 6 arms for rotarod test. For example, for infarct volume evaluation, the biggest effect sizes were 10.48 and 16.49 in one study by Chen *et al*.^29^. They compared transplantation of NSCs modified by glial cell line-derived neurotrophic factor (GDNF) gene to native NSCs transplantation after ischemia stroke. They found that the total lesion volume in NSCs and GDNF/NSCs groups was significantly reduced compared to controls. However, significant difference effect in GDNF/NSCs compared to NSCs group was only observed at first week. At 7th week, the lesion volume in both NSCs and GDNF/NSCs group was nearly less than 10% and almost recovered. Nevertheless, they have neither randomized grouping or allocation concealment, nor enough number in a group. Other improper interventions also have the similar defects such as non-randomized allocation concealment or insufficient experimental number (*e.g.* each group number less than 6). These defects could result in low study quality and more publication bias, leading to the overestimation of the effects in NSCs treatment.

Our study is observational rather than experimental which is the main limitation. All findings are hypothesis and only the association of studies is reported. Although our search strategy was exhaustive, it is also possible that some published studies were missed. Moreover, our data set is small. Although 2524 publications were identified by electronic searching, there were only 37 publications met our criteria. Among these, 36 studies reported functional (neurobehavioral) outcome alone, 22 reported structural (infarct volume) outcome alone and 21 reported both structural and functional outcome. In total 54 experiment arms, there were only 1009 animals that reported functional outcome and 639 animals reported structural outcome. Furthermore, we found that some interventions were not appropriate. Therefore we removed 9 or 8 treatment arms in infarct volume or mNSS analysis respectively. The low quality studies normally overstate efficacy. Due to the possible use of low quality studies in our analysis, our study may have same overestimated NSCs treatment effects. Nevertheless, our study is likely to have reported the prime tendency in the field of NSCs treatment and represents the complete review for the use of NSCs in experimental ischemia stroke.

In brief, our findings suggest that, for best functional outcome, homologous cells should be used (experiment in mouse or rat, mNSCs > rNSCs > hNSCs). Moreover, these cells should be given within the first 24 hours poststroke or no more than 72 hours. The allogeneic cells usually have less immunological rejection than xenogenic cells. We did not find the direct correlation of the dose of intervention and the route of delivery to any outcome. It may be due to the small sample size of studies. Furthermore, most studies used same intervention route of stereotaxic transplantation. Therefore the dose and the route appear to be less important. These findings could provide some guidance for the design of clinical trials, and give some measures to improve internal validity and reduce publication bias^30^. The clinical translational NSCs therapy for human should be directed more carefully than in rodents, the safety of NSCs transplantation have to be recorded in clinical trials of human with cerebrovascular diseases.

The current meta-analysis of preclinical studies of NSCs therapy for ischemic stroke demonstrated significant and favorable effects on behavioral and structural outcomes. Furthermore, it provides some guidance for future clinical trials. Firstly, the internal (study quality) and external (publication bias) validity should be improved to provide solid foundation for clinical translational trials. Secondly, a number of factors, such as cells species source, time of NSCs administration after stroke, dose of administration and route of administration, should be taken into consideration to support clinical translational trials. Finally, the current meta-analysis and systematic review have suggested some useful tools for the further translational studies of NSCs in human ischemic stroke. In the future NSCs clinical treatment (*e.g.* human ischemia stroke)^31^, it is important to consider the stem cell species source, transplantation time-window, long term effects, risk assessment, as well as the stem cell survival stage and neural functional repair gains.

## Additional Information

**How to cite this article**: Chen, L. *et al*. Meta-Analysis and Systematic Review of Neural Stem Cells therapy for experimental ischemia stroke in preclinical studies. *Sci. Rep.* **6**, 32291; doi: 10.1038/srep32291 (2016).

## Supplementary Material

Supplementary Information

## Figures and Tables

**Table 1:** Effect size for NSCs administration in studies of animal ischemic stroke.

Measure	All studies			Studies after adjustment			
	Mean (Effect Size)	95% CI	No.	ES >3 arms No.	Mean (ES)	95% CI	No.
Infarct volume	1.45	1.01–1.88	34	9	0.84	0.51–1.61	25
mNSS	2.00	1.55–2.46	28	8	1.35	1.04–1.66	20
Rotarod test	2.48	1.65–3.32	19	6	1.84	1.38–2.30	11
Cylinder test	0.61	0.17–1.04	9	0	—	—	—

Note: For each effect size, the 95% CI does not cross zero and the *p* value for each effect was <0.01, indicating that results favor NSCs. ES = Effect Size, CI = Confidence Interval.

**Table 2:** Bivariate analysis between clinical variables and NSCs effect size.

Effect Size for Infarct Volume			Effect Size for mNSS		
Correlations with	r	*p*	Correlations with	r	*p*
Time NSCs administered poststroke	−0.472	0.017	Time NSCs administered poststroke	−0.508	0.022
Use of immunosuppression	—	0.048	Use of immunosuppression	—	0.038
Type of NSC source (human <rat <mouse)	0.465	0.019			

Note: For the *p* value for effect was <0.05 and r >0.3, indicating that results for clinical variables had a certain correlation to effect size.
