# Supplementary material for: Meta-Analysis and Systematic Review of Neural Stem Cells therapy for experimental ischemia stroke in preclinical studies
Source: Sci Rep. 2016 Aug 24;6:32291. doi: 10.1038/srep32291 (PMC4995377; doi:10.1038/srep32291)
Supplement: Supplementary Information [file srep32291-s1.pdf]

Online Supplements

Meta-Analysis and Systematic Review of Neural Stem Cells therapy for experimental ischemia stroke in preclinical studies

Lukui Chen[1*†], Guilong Zhang[1†], Yuchun Gu[2], Xiaoyuan Guo[1]

[1]Department of Neurosurgery, Zhongda Hospital, School of Medicine, Southeast University, Nanjing, China

[2]Molecular Pharmacology Laboratory, Institute of Molecular Medicine, Peking University, Beijing, 100871, China

[†]These two authors have contributed equally to this study.

[*]Corresponding Author:

Lukui Chen, Department of Neurosurgery, Zhongda Hospital Southeast University, 87 Dingjiaqiao, Gulou District, Nanjing, 210009, China; Tel: 86-25-83262271; Fax: 86-25-83272011;

Email: neuro_clk@hotmail.com

Supplementary Fig. 1 Effect sizes of all studies (no excluded)

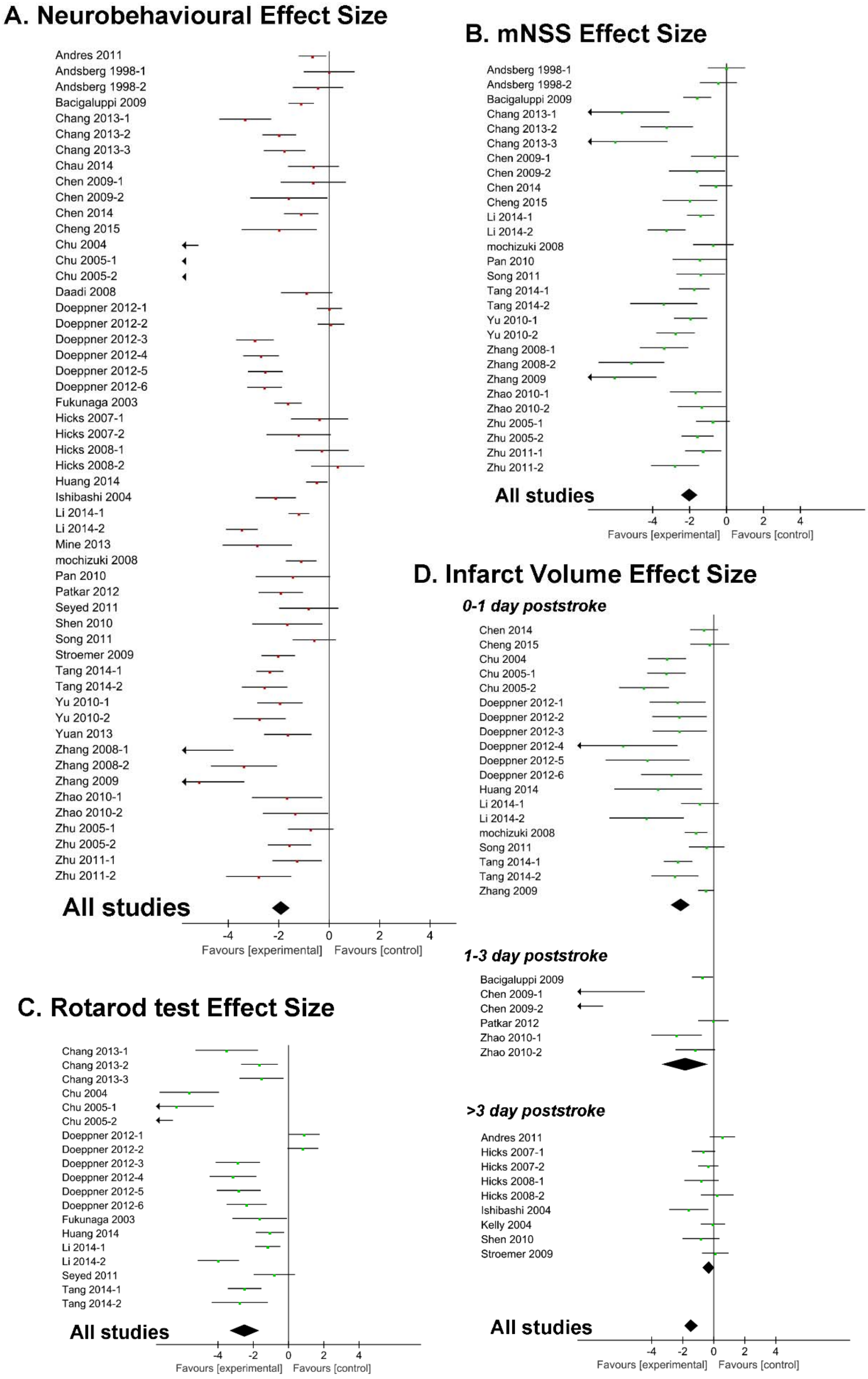

Supplementary Table 1 Search Strategy (Take PubMed for instance)

**Search**	(((((((("Brain Ischemia"[Mesh]) OR ((brain ischaemia[Title/Abstract] OR stroke*[Title/Abstract] OR cerebrovascular stroke[Title/Abstract] OR apoplexy[Title/Abstract] OR cerebral stroke[Title/Abstract] OR cerebrovascular accident[Title/Abstract] OR acute cerebrovascular accident[Title/Abstract] OR cerebrovascular apoplexy[Title/Abstract] OR acute stroke[Title/Abstract] OR ischemic stroke[Title/Abstract] OR ischaemic stroke[Title/Abstract] OR acute ischemic stroke[Title/Abstract] OR acute ischaemic stroke[Title/Abstract] OR vascular brain accident[Title/Abstract] OR middle cerebral artery[Title/Abstract] OR middle cerebral artery infarction[Title/Abstract] OR middle cerebral artery occlusion[Title/Abstract] OR MCA occlusion[Title/Abstract] OR MCA[Title/Abstract] OR MCAO[Title/Abstract] OR anterior cerebral artery[Title/Abstract] OR ACA[Title/Abstract] OR anterior cerebral artery occlusion[Title/Abstract] OR ACAO[Title/Abstract] OR focal cerebral ischemia[Title/Abstract] OR focal ischemia[Title/Abstract])))) AND (("Neural Stem Cells"[Mesh]) OR ((neural stem*[Title/Abstract] OR NSC*[Title/Abstract] OR neural precursor cells[Title/Abstract] OR neural precursor*[Title/Abstract] OR NPC*[Title/Abstract] OR nerve stem cell*[Title/Abstract] OR neuronal stem cell*[Title/Abstract]))))) AND ((animals[MeSH Terms]) OR ((mammals[Title/Abstract] OR primates [Title/Abstract] OR mice[Title/Abstract] OR Rats[Title/Abstract] OR Mouse[Title/Abstract]))))) NOT (((((heart[Title/Abstract] OR bone[Title/Abstract] OR eye[Title/Abstract] OR lung[Title/Abstract] OR kidney[Title/Abstract] OR liver[Title/Abstract] OR renal[Title/Abstract] OR intestine*[Title/Abstract] OR spinal[Title/Abstract] OR pulmonary[Title/Abstract] OR hepatic[Title/Abstract] OR global[Title/Abstract]))) OR ((coronary[Title/Abstract] OR myocardial[Title/Abstract]))))) NOT ((AD[Title/Abstract] OR PD[Title/Abstract] OR Alzheimer*[Title/Abstract] OR Parkinson* [Title/Abstract] OR epilepsy[Title/Abstract] OR MS[Title/Abstract] OR Multiple Sclerosis[Title/Abstract]))

Supplementary Fig. 2 Flow diagram showing summary of study selection procedure

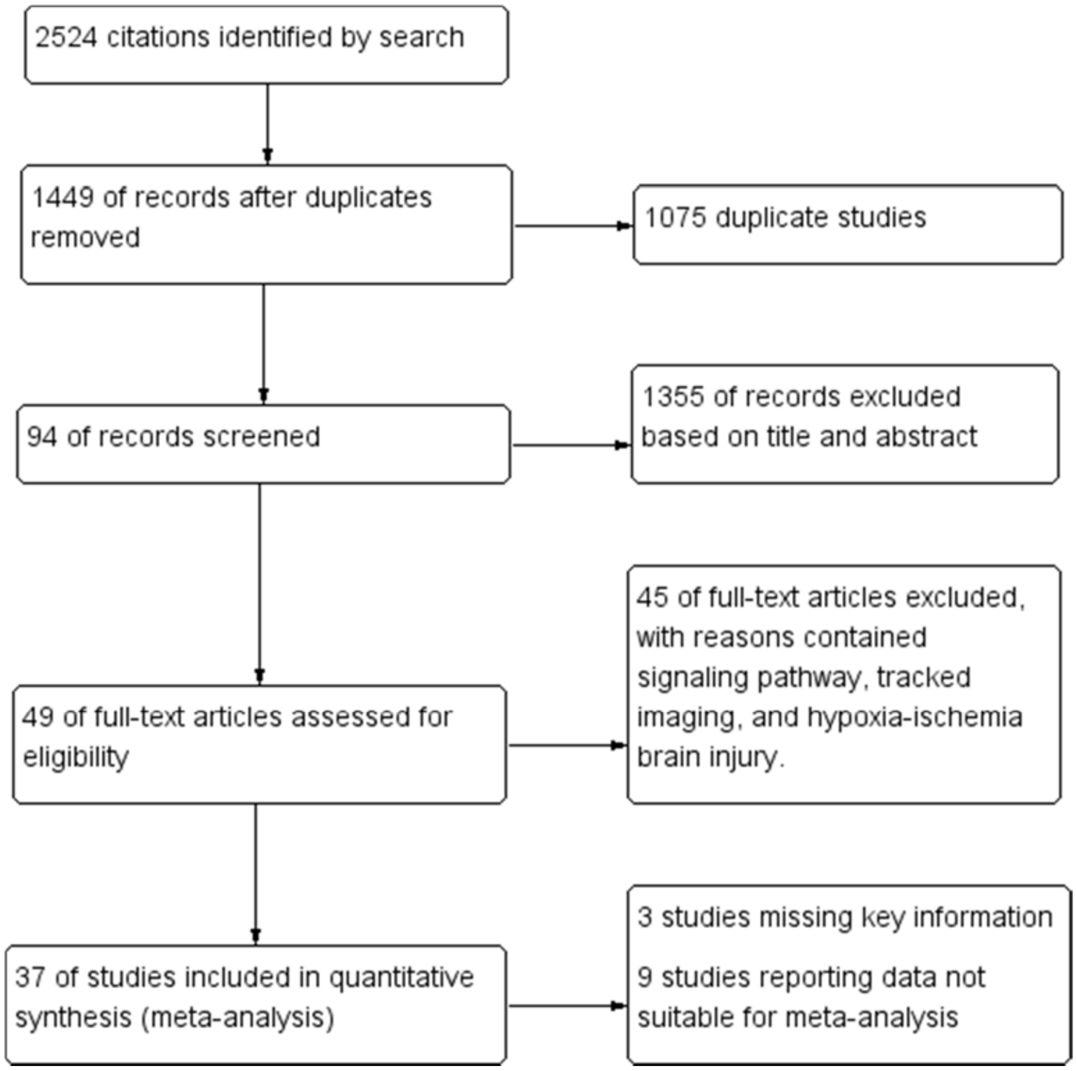

Supplementary Table 2 Study Characteristic Report

Author	Year	Intervention	Species	Type of Cerebral Ischemia	No. of Animals	Dose Range(cells)	Time of Admin(min)	Route of Delivery	Anaesthetic
Andsberg G[1]	1998-1	rat NSC	Rat	Transient MCAO	10	900000	-10080	Stereotactic	Halothane
	1998-2	rat NGF-NSC	Rat	Transient MCAO	10	900000	-10080	Stereotactic	Halothane
Fukunaga A[2]	2003	rat NSC	Rat	Transient MCAO	5	55000	30240	Stereotactic	Halothane
Chu K[3]	2004	human NSC	Rat	Transient MCAO	30	5000000	1440	tail vein	none
Ishibashi S[4]	2004	human NSPC	Gerbil	Transient MCAO	7	500000	5760	Stereotactic	Isoflurane
Kelly S[5]	2004	human NSC	Rat	Transient MCAO	13	300000	10080	Stereotactic	Isoflurane
Chu K[6]	2005-1	human NSC	Rat	Transient MCAO	12	5000000	1440	tail vein	none
	2005-2	hNSC-VEGF	Rat	Transient MCAO	12	5000000	1440	tail vein	none
Zhu W[7]	2005	rat NSC	Rat	Transient MCAO	10	200000	4320	Stereotactic	ketamine
	2005	rat NSC-VEGF	Rat	Transient MCAO	10	200000	4320	Stereotactic	ketamine
Hicks A[8]	2007-1	mouse NSC-ST	Rat	Transient MCAO	10	800000	10080	Stereotactic	Isoflurane
	2007-2	mouse NSC-EE	Rat	Transient MCAO	15	800000	10080	Stereotactic	Isoflurane
Daadi M[9]	2008	Human ESC-NSC	Rat	Transient MCAO	10	100000	10080	Stereotactic	Unknown
Hicks A[10]	2008-1	mouse NSC-ST	Rat	Transient MCAO	13	800000	10080	Stereotactic	Isoflurane
	2008-2	mouse NSC-EE	Rat	Transient MCAO	13	800000	10080	Stereotactic	Isoflurane
Mochizuki N[11]	2008	rat NPC	Rat	Thrombotic	17	100000	unknow	Stereotactic	Pentobarbital
Zhang Z[12]	2008-1	rat NSC	Rat	Transient MCAO	12	200000	10080	Stereotactic	Chloral Hydrate
	2008-2	rat NSC-hNT3	Rat	Transient MCAO	12	200000	10080	Stereotactic	Chloral Hydrate
Bacigaluppi M[13]	2009	mouse NSC	mice	Transient MCAO	18	1000000	4320	tail vein	none
Chen B[14]	2009-1	rat NSC	Rat	Transient MCAO	10	500000	4320	Stereotactic	Pentobarbital
	2009-2	rat NSC-GDNF	Rat	Transient MCAO	10	500000	4320	Stereotactic	Pentobarbital

Author	Year	Intervention	Species	Type of Cerebral Ischemia	No. of Animals	Dose Range(cells)	Time of Admin(min)	Route of Delivery	Anaesthetic
Stroemer P[15]	2009	human NSC	Rat	Transient MCAO	10	450000	40320	Stereotactic	halothane
Zhang P[16]	2009	human NSC	Rat	Permanent	30	50000	1440	Stereotactic	Pentobarbital
Pan[17]	2010	hNSC	Rat	Transient MCAO	20	3000000	1440	Stereotactic	Unknown
Shen C[18]	2010	rat NSC	Rat	Transient MCAO	12	10000000	20160	femoral veins	Isoflurane
Yu H[19]	2010-1	rat NSC	Rat	Transient MCAO	18	15000	1440	Stereotactic	Chloral Hydrate
	2010-2	rat NSC-coll	Rat	Transient MCAO	18	15000	1440	Stereotactic	Chloral Hydrate
Zhao Y[20]	2010-1	rat NSC	Rat	Transient MCAO	12	50000	2880	Stereotactic	ketamine
	2010-2	rat NSC-wort	Rat	Transient MCAO	12	50000	2880	Stereotactic	ketamine
Andres R[21]	2011	human NSC	nude rat	Permanent	12	300000	10080	Stereotactic	Isoflurane
Seyed J[22]	2011	rat NSC	Rat	Transient MCAO	8	10000000	4320	lumbar puncture	Chloral Hydrate
Song M[23]	2011	human NSC	Rat	Transient MCAO	6	4000000	1440	tail vein	none
Zhu J[24]	2011-1	rat NSC	Rat	Transient MCAO	10	1000000	4320	Stereotactic	Chloral Hydrate
	2011-2	rat NSC-BDNF	Rat	Transient MCAO	20	1000000	4320	Stereotactic	Chloral Hydrate
	2012-1	mouse NSC-ic	mice	Transient MCAO	14	500000	360	Stereotactic	ketamine
	2012-2	mouse NSC-ic-TAT-HA	mice	Transient MCAO	14	500000	360	Stereotactic	ketamine
Doeppner T[25]	2012-3	mouse NSC-ic-TAT-HS	mice	Transient MCAO	14	500000	360	Stereotactic	ketamine
	2012-4	mouse NSC-iv	mice	Transient MCAO	14	1000000	360	femoral vein	none
	2012-5	mouse NSC-iv-TAT-HA	mice	Transient MCAO	14	1000000	360	femoral vein	none
	2012-6	mouse NSC-ivTAT-HS	mice	Transient MCAO	14	1000000	360	femoral vein	none
Patkar S[26]	2012	mouse NSC	mice	Transient MCAO	8	50000	2880	Stereotactic	Isoflurane
Chang D J[27]	2013-1	hNSC-BDNF	Rat	Transient MCAO	8	400000	10080	Stereotactic	Unknown
Chang D J[28]	2013-2	hipsc-NPC	Rat	Transient MCAO	10	200000	10080	Stereotactic	Unknown
Chang D J[29]	2013-3	hESC-NPC	Rat	Transient MCAO	8	200000	10080	Stereotactic	ketamine

Author	Year	Intervention	Species	Type of Cerebral Ischemia	No. of Animals	Dose Range(cells)	Time of Admin(min)	Route of Delivery	Anaesthetic
Mine Y[30]	2013	hNPSC	nude rat	Transient MCAO	18	200000	2880	Stereotactic	Isoflurane
Yuan T[31]	2013	hipsc-NPC	Rat	Transient MCAO	5	1000000	unknow	Stereotactic	Unknown
Chau M J[32]	2014	mouse-ips-NPC	Rat	Permanent	24	400000	10080	Stereotactic	Chloral Hydrate
Chen L[33]	2014	rat NSC	rat	Transient MCAO	10	80000	1440	Stereotactic	Chloral Hydrate
Huang L[34]	2014	hNSC	mice	Transient MCAO	18	100000	1440	Stereotactic	Isoflurane
Li J[35]	2014-1	mouse NSC-irrFCs	Rat	Transient MCAO	18	1000000	1440	Stereotactic	Unknown
	2014-2	mouse NSC-VPC	Rat	Transient MCAO	18	1000000	1440	Stereotactic	Unknown
Tang Y[36]	2014-1	mice NSC	Rat-Y	Transient MCAO	16	1000000	1440	Stereotactic	ketamine
	2014-2	mice NSC	Rat-A	Transient MCAO	7	1000000	1440	Stereotactic	ketamine
Cheng Y[37]	2015	rat NSC	Rat	Transient MCAO	18	5000000	1440	tail vein	none

Abbreviation: NGF = Nerve Growth Factor, VEGF = Vascular Endothelial Growth Factor, EE = Enriched Environment, ST = Standard housing, hNT-3 = human neurotrophin-3, GDNF = Glial cell line-derived Neurotrophic Factor, Coll = Collagen type I, Wort = Wortmannin, BDNF = Brain-derived Neurotrophic Factor, TAT-HA = TAT-hemagglutinin, TAT-HS = TAT-heat shock protein 70, ic = intracranial injection, iv = intravenous injection, irrFCs = gamma-irradiated embryonic fibroblast cells, VPC = Vascular Progenitor cells, Y = young, A = aged, ESC = embryonic stem cell, ips = induced pluripotent stem cells.

Supplementary Table 3 Outcome measures report

Author	Year	Intervention	Structural Outcome	Functional Outcome	mNSS	Rotarod test	Cylinder test
Andsberg G	1998-1	rat NSC		Neurobehavioural	√		
	1998-2	rat NGF-NSC		Neurobehavioural	√		
Fukunaga A	2003	rat NSC		Neurobehavioural		√	
Chu K	2004	human NSC	Infarct Volume	Neurobehavioural		√	
Ishibashi S	2004	human NSPC	Infarct Volume	Neurobehavioural			
Kelly S	2004	human NSC	Infarct Volume				
Chu K	2005-1	human NSC	Infarct Volume	Neurobehavioural		√	
	2005-2	hNSC-VEGF	Infarct Volume	Neurobehavioural		√	
Zhu W	2005-1	rat NSC		Neurobehavioural	√		
	2005-2	rat NSC-VEGF		Neurobehavioural	√		
Hicks A	2007-1	mouse NSC-ST	Infarct Volume	Neurobehavioural			√
	2007-2	mouse NSC-EE	Infarct Volume	Neurobehavioural			√
Daadi M	2008	human ESC-NSC		Neurobehavioural			√
Hicks A	2008-1	mouse NSC-ST	Infarct Volume	Neurobehavioural			√
	2008-2	mouse NSC-EE	Infarct Volume	Neurobehavioural			√
Mochizuki N	2008	rat NPC	Infarct Volume	Neurobehavioural	√		
Zhang Z	2008-1	rat NSC		Neurobehavioural	√		
	2008-2	rat NSC-hNT3		Neurobehavioural	√		
Bacigaluppi M	2009	mouse NSC	Infarct Volume	Neurobehavioural	√		
Chen B	2009-1	rat NSC	Infarct Volume	Neurobehavioural	√		
	2009-2	rat NSC-GDNF	Infarct Volume	Neurobehavioural	√		

Author	Year	Intervention	Structural Outcome	Functional Outcome	mNSS	Rotarod test	Cylinder test
Stroemer P	2009	human NSC	Infarct Volume	Neurobehavioural			
Zhang P	2009	human NSC	Infarct Volume	Neurobehavioural	√		
Pan	2010	human NSC		Neurobehavioural	√		
Shen C	2010	rat NSC	Infarct Volume	Neurobehavioural			
Yu H	2010-1	rat NSC		Neurobehavioural	√		
	2010-2	rat NSC-coll		Neurobehavioural	√		
Zhao Y	2010-1	rat NSC	Infarct Volume	Neurobehavioural	√		
	2010-2	rat NSC-wort	Infarct Volume	Neurobehavioural	√		
Andres R	2011	human NSC	Infarct Volume	Neurobehavioural			√
Seyed J	2011	rat NSC		Neurobehavioural		√	
Song M	2011	human NSC	Infarct Volume	Neurobehavioural	√		√
Zhu J	2011-1	rat NSC		Neurobehavioural	√		
	2011-2	rat NSC-BDNF		Neurobehavioural	√		
	2012-1	mouse NSC-ic	Infarct Volume	Neurobehavioural		√	
	2012-2	mouse NSC-ic-TAT-HA	Infarct Volume	Neurobehavioural		√	
Doeppner T	2012-3	mouse NSC-ic-TAT-HS	Infarct Volume	Neurobehavioural		√	
	2012-4	mouse NSC-iv	Infarct Volume	Neurobehavioural		√	
	2012-5	mouse NSC-iv-TAT-HA	Infarct Volume	Neurobehavioural		√	
	2012-6	mouse NSC-ivTAT-HS	Infarct Volume	Neurobehavioural		√	
Patkar S	2012	mouse NSC	Infarct Volume	Neurobehavioural			√
Chang D J	2013-1	hNSC-BDNF		Neurobehavioural	√	√	
Chang D J	2013-2	hipsc-NPC		Neurobehavioural	√	√	
Chang D J	2013-3	hESC-NPC		Neurobehavioural	√	√	

Author	Year	Intervention	Structural Outcome	Functional Outcome	mNSS	Rotarod test	Cylinder test
Mine Y	2013	human NPSC		Neurobehavioural			√
Yuan T	2013	hipsc-NPC		Neurobehavioural			
Chau M J	2014	mouse-ips-NPC		Neurobehavioural			
Chen L	2014	rat NSC	Infarct Volume	Neurobehavioural	√		
Huang L	2014	human NSC	Infarct Volume	Neurobehavioural		√	
Li J	2014-1	mouse NSC-irrFCs	Infarct Volume	Neurobehavioural	√	√	
	2014-2	mouse NSC-VPC	Infarct Volume	Neurobehavioural	√	√	
Tang Y	2014-1	mice NSC	Infarct Volume	Neurobehavioural	√	√	
	2014-2	mice NSC	Infarct Volume	Neurobehavioural	√	√	
Cheng Y	2015	rat NSC	Infarct Volume	Neurobehavioural	√		

Supplementary Table 4 Study Quality Report

Author	Year	(1)	(2)	(3)	(4)	(5)	(6)	(7)	(8)	(9)	(10)	Quality Score
Andsberg G	1998	1	1	1	1	1	1	0	0	0	0	6
Fukunaga A	2003	1	1	0	0	0	1	0	0	0	0	3
Chu K	2004	1	1	1	1	0	1	0	0	1	0	6
Ishibashi S	2004	1	0	1	0	1	1	0	0	1	0	5
Kelly S	2004	1	1	0	0	0	1	0	0	1	0	4
Chu K	2005	1	0	0	0	1	1	0	0	1	0	4
Zhu W	2005	1	1	0	0	1	1	0	0	1	0	5
Hicks A	2007	1	1	1	1	1	1	0	0	1	0	7
Daadi M	2008	1	0	0	0	0	1	0	0	1	0	3
Hicks A	2008	1	1	1	1	0	1	0	0	1	0	6
Mochizuki N	2008	1	0	0	0	0	1	0	0	1	0	3
Zhang Z	2008	1	1	1	1	1	1	0	0	1	0	7
Bacigaluppi M	2009	1	1	1	1	0	1	0	0	1	0	6
Chen B	2009	1	1	0	0	1	1	0	0	1	0	5
Stroemer P	2009	1	1	0	0	1	1	0	0	1	0	5
Zhang P	2009	1	1	0	0	1	1	0	0	1	0	5
Pan	2010	1	0	1	1	0	0	0	0	1	0	4
Shen C	2010	1	1	1	1	0	1	0	0	1	0	6
Yu H	2010	1	1	1	1	1	1	0	0	1	0	7
Zhao Y	2010	1	0	1	1	0	1	0	0	1	1	6
Andres R	2011	1	0	0	0	1	1	0	0	1	0	4
Seyed J	2011	1	1	0	0	1	1	0	0	1	0	5

Author	Year	(1)	(2)	(3)	(4)	(5)	(6)	(7)	(8)	(9)	(10)	Quality Score
Song M	2011	1	1	0	0	1	1	0	0	1	1	6
Zhu J	2011	1	0	1	1	0	1	0	0	1	1	6
Doeppner T	2012	1	1	0	0	1	1	0	0	1	1	6
Patkar S	2012	1	1	0	0	1	1	0	0	1	0	5
Chang D J	2013	1	0	0	0	0	0	0	0	1	1	3
Chang D J	2013	1	0	0	0	0	0	0	0	1	1	3
Chang D J	2013	1	1	0	0	0	1	0	0	1	1	5
Mine Y	2013	1	0	0	0	1	1	0	0	1	0	4
Yuan T	2013	1	0	1	0	0	0	0	0	1	1	4
Chau M J	2014	1	0	0	0	0	1	0	0	1	1	4
Chen L	2014	1	0	1	0	1	1	0	0	1	0	5
Huang L	2014	1	1	0	0	0	1	0	0	1	1	5
Li J	2014	1	0	1	0	1	0	0	0	1	0	4
Tang Y	2014	1	1	1	0	1	1	0	0	1	1	7
Cheng Y	2015	1	1	1	0	1	1	0	0	1	1	7

Study quality items:

(1) Publication in a peer-reviewed journal

(2) Statement describing control of temperature

(3) Statement of randomisation to treatment group

(4) Statement of allocation concealment

(5) Statement of blinded assessment of outcome

(6) Avoidance of anaesthetics with known marked intrinsic neuroprotective properties

(7) Use of animals with relevant co-morbidities

(8) Statement of sample size calculation

(9) Statement of compliance with animal welfare regulations

(10) Statement of whether or not the authors had possible conflicts of interest

References

1. Andsberg, G., Kokaia, Z., Bjorklund, A., Lindvall, O. & Martinez-Serrano, A. Amelioration of ischaemia-induced neuronal death in the rat striatum by NGF-secreting neural stem cells. *Eur J Neurosci* **10**, 2026-36 (1998).
2. Fukunaga, A., Kawase, T. & Uchida, K. Functional recovery after simultaneous transplantation with neuro-epithelial stem cells and adjacent mesenchymal tissues into infarcted rat brain. *Acta Neurochirurgica* **145**, 473-481 (2003).
3. Chu, K. et al. Human neural stem cells improve sensorimotor deficits in the adult rat brain with experimental focal ischemia. *Brain Res* **1016**, 145-53 (2004).
4. Ishibashi, S. et al. Human neural stem/progenitor cells, expanded in long-term neurosphere culture, promote functional recovery after focal ischemia in Mongolian gerbils. *J Neurosci Res* **78**, 215-23 (2004).
5. Kelly, S. et al. Transplanted human fetal neural stem cells survive, migrate, and differentiate in ischemic rat cerebral cortex. *Proc Natl Acad Sci U S A* **101**, 11839-44 (2004).
6. Chu, K. et al. Combined treatment of vascular endothelial growth factor and human neural stem cells in experimental focal cerebral ischemia. *Neurosci Res* **53**, 384-90 (2005).
7. Zhu, W. et al. Transplantation of vascular endothelial growth factor-transfected neural stem cells into the rat brain provides neuroprotection after transient focal cerebral ischemia. *Neurosurgery* **57**, 325-33; discussion 325-33 (2005).
8. Hicks, A.U. et al. Enriched environment enhances transplanted subventricular zone stem cell migration and functional recovery after stroke. *Neuroscience* **146**, 31-40 (2007).
9. Daadi, M.M., Maag, A.L. & Steinberg, G.K. Adherent self-renewable human embryonic stem cell-derived neural stem cell line: functional engraftment in experimental stroke model. *PLoS One* **3**, e1644 (2008).
10. Hicks, A.U., MacLellan, C.L., Chernenko, G.A. & Corbett, D. Long-term assessment of enriched housing and subventricular zone derived cell transplantation after focal ischemia in rats. *Brain Research* **1231**, 103-112 (2008).
11. Mochizuki, N. et al. Injection of neural progenitor cells improved learning and memory dysfunction after cerebral ischemia. *Exp Neurol* **211**, 194-202 (2008).
12. Zhang, Z.H. et al. Transplantation of neural stem cells modified by human neurotrophin-3 promotes functional recovery after transient focal cerebral ischemia in rats. *Neurosci Lett* **444**, 227-30 (2008).
13. Bacigaluppi, M. et al. Delayed post-ischaemic neuroprotection following systemic neural stem cell transplantation involves multiple mechanisms. *Brain* **132**, 2239-2251 (2009).
14. Chen, B. et al. Neuroprotective effect of grafting GDNF gene-modified neural stem cells on cerebral ischemia in rats. *Brain Res* **1284**, 1-11 (2009).
15. Stroemer, P. et al. The neural stem cell line CTX0E03 promotes behavioral recovery and endogenous neurogenesis after experimental stroke in a dose-dependent fashion. *Neurorehabil Neural Repair* **23**, 895-909 (2009).
16. Zhang, P. et al. Human neural stem cell transplantation attenuates apoptosis and improves neurological functions after cerebral ischemia in rats. *Acta Anaesthesiol Scand* **53**, 1184-91 (2009).
17. Pan, F. et al. Stem cell transplantation for treatment of cerebral ischemia in rats Effects of

human umbilical cord blood stem cells and human neural stem cells. *Neural Regeneration Research* **5**, 485-490 (2010).

18. Shen, C.C. et al. Intravenous implanted neural stem cells migrate to injury site, reduce infarct volume, and improve behavior after cerebral ischemia. *Curr Neurovasc Res* **7**, 167-79 (2010).
19. Yu, H. et al. Combinated transplantation of neural stem cells and collagen type I promote functional recovery after cerebral ischemia in rats. *Anat Rec (Hoboken)* **293**, 911-7 (2010).
20. Zhao, Y., Yao, S. & Wang, S. Neural stem cell transplantation in the hippocampus of rats with cerebral ischemia/reperfusion injury Activation of the phosphatidylinositol-3 kinase/Akt pathway and increased brain-derived neurotrophic factor expression. *Neural Regeneration Research* **5**, 1605-1610 (2010).
21. Andres, R.H. et al. Human neural stem cells enhance structural plasticity and axonal transport in the ischaemic brain. *Brain* **134**, 1777-89 (2011).
22. Seyed Jafari, S.S., Ali Aghaei, A., Asadi-Shekaari, M., Nematollahi-Mahani, S.N. & Sheibani, V. Investigating the effects of adult neural stem cell transplantation by lumbar puncture in transient cerebral ischemia. *Neurosci Lett* **495**, 1-5 (2011).
23. Song, M. et al. Effects of duplicate administration of human neural stem cell after focal cerebral ischemia in the rat. *Int J Neurosci* **121**, 457-61 (2011).
24. Zhu, J.M. et al. Functional recovery after transplantation of neural stem cells modified by brain-derived neurotrophic factor in rats with cerebral ischaemia. *J Int Med Res* **39**, 488-98 (2011).
25. Doeppner, T.R. et al. Transduction of neural precursor cells with TAT-heat shock protein 70 chaperone: therapeutic potential against ischemic stroke after intrastriatal and systemic transplantation. *Stem Cells* **30**, 1297-310 (2012).
26. Patkar, S., Tate, R., Modo, M., Plevin, R. & Carswell, H.V. Conditionally immortalised neural stem cells promote functional recovery and brain plasticity after transient focal cerebral ischaemia in mice. *Stem Cell Res* **8**, 14-25 (2012).
27. Chang, D.J. et al. Therapeutic effect of BDNF-overexpressing human neural stem cells (HB1.F3.BDNF) in a rodent model of middle cerebral artery occlusion. *Cell Transplant* **22**, 1441-52 (2013).
28. Chang, D.J. et al. Therapeutic potential of human induced pluripotent stem cells in experimental stroke. *Cell Transplant* **22**, 1427-40 (2013).
29. Chang, D.J. et al. Contralaterally transplanted human embryonic stem cell-derived neural precursor cells (ENStem-A) migrate and improve brain functions in stroke-damaged rats. *Exp Mol Med* **45**, e53 (2013).
30. Mine, Y. et al. Grafted human neural stem cells enhance several steps of endogenous neurogenesis and improve behavioral recovery after middle cerebral artery occlusion in rats. *Neurobiol Dis* **52**, 191-203 (2013).
31. Yuan, T. et al. Human induced pluripotent stem cell-derived neural stem cells survive, migrate, differentiate, and improve neurologic function in a rat model of middle cerebral artery occlusion. *Stem Cell Res Ther* **4**, 73 (2013).
32. Chau, M.J. et al. iPSC Transplantation increases regeneration and functional recovery after ischemic stroke in neonatal rats. *Stem Cells* **32**, 3075-87 (2014).
33. Chen, L. et al. The role of exogenous neural stem cells transplantation in cerebral ischemic stroke. *J Biomed Nanotechnol* **10**, 3219-30 (2014).

34. Huang, L., Wong, S., Snyder, E.Y., Hamblin, M.H. & Lee, J.P. Human neural stem cells rapidly ameliorate symptomatic inflammation in early-stage ischemic-reperfusion cerebral injury. *Stem Cell Res Ther* **5**, 129 (2014).
35. Li, J. et al. Neurovascular recovery via co-transplanted neural and vascular progenitors leads to improved functional restoration after ischemic stroke in rats. *Stem Cell Reports* **3**, 101-14 (2014).
36. Tang, Y. et al. Neural stem cell protects aged rat brain from ischemia-reperfusion injury through neurogenesis and angiogenesis. *J Cereb Blood Flow Metab* **34**, 1138-47 (2014).
37. Cheng, Y. et al. Intravenously delivered neural stem cells migrate into ischemic brain, differentiate and improve functional recovery after transient ischemic stroke in adult rats. *Int J Clin Exp Pathol* **8**, 2928-36 (2015).
